# Risk of node metastasis of sentinel lymph nodes detected in level II/III of the axilla by single-photon emission computed tomography/computed tomography

**DOI:** 10.3892/etm.2014.1968

**Published:** 2014-09-15

**Authors:** HIROAKI SHIMA, GORO KUTOMI, FUKINO SATOMI, HIDEKI MAEDA, TOMOKO TAKAMARU, HIDEKAZU KAMESHIMA, TOSEI OMURA, MITSURU MORI, MASAMITSU HATAKENAKA, TADASHI HASEGAWA, KOICHI HIRATA

**Affiliations:** 1 Department of Surgery, Surgical Oncology and Science, Sapporo Medical University, Sapporo 060-8543, Japan; 2 Department of Breast Surgery Oncology, Showa University School of Medicine, Tokyo 142-8666, Japan; 3 Department of Surgery, Higashi Sapporo Hospital, Sapporo 003-8585, Japan; 4 Department of Public Health, Sapporo Medical University, Sapporo 060-8556, Japan; 5 Department of Diagnostic Radiology, Sapporo Medical University, Sapporo 060-8543, Japan; 6 Department of Surgical Pathology, Sapporo Medical University, Sapporo 060-8543, Japan

**Keywords:** breast cancer, sentinel node biopsy, single-photon emission computed tomography/computed tomography, atypical lymphatic drainage, level II, level III

## Abstract

In breast cancer, single-photon emission computed tomography/computed tomography (SPECT/CT) shows the exact anatomical location of sentinel nodes (SN). SPECT/CT mainly exposes axilla and partly exposes atypical sites of extra-axillary lymphatic drainage. The mechanism of how the atypical hot nodes are involved in lymphatic metastasis was retrospectively investigated in the present study, particularly at the level II/III region. SPECT/CT was performed in 92 clinical stage 0-IIA breast cancer patients. Sentinel lymph nodes are depicted as hot nodes in SPECT/CT. Patients were divided into two groups: With or without hot node in level II/III on SPECT/CT. The existence of metastasis in level II/III was investigated and the risk factors were identified. A total of 12 patients were sentinel lymph node biopsy metastasis positive and axillary lymph node dissection (ALND) was performed. These patients were divided into two groups: With and without SN in level II/III, and nodes in level II/III were pathologically proven. In 11 of the 92 patients, hot nodes were detected in level II/III. There was a significant difference in node metastasis depending on whether there were hot nodes in level II/III (P=0.0319). Multivariate analysis indicated that the hot nodes in level II/III and lymphatic invasion were independent factors associated with node metastasis. There were 12 SN-positive patients followed by ALND. In four of the 12 patients, hot nodes were observed in level II/III. Two of the four patients with hot nodes depicted by SPECT/CT and metastatic nodes were pathologically evident in the same lesion. Therefore, the present study indicated that the hot node in level II/III as depicted by SPECT/CT may be a risk of SN metastasis, including deeper nodes.

## Introduction

Currently, sentinel lymph node biopsy (SNB) has become standard practice to avoid axillary lymph node dissection (ALND) in patients with clinically node-negative breast cancer (1). Breast cancer lymphatic drainage patterns are fairly predictable, with the vast majority of lesions exhibiting primary drainage to axillary lymph nodes (2). In particular, it has been reported that the majority of drained sentinel nodes (SN) are in the level I nodes in the axilla (3). However, there are certain studies mentioning the potential to drain to an SN in level II and III nodes or an SN outside the axilla (3).

Single-photon emission computed tomography/computed tomography (SPECT/CT) shows the exact anatomical location of SN (4). The first large study on SPECT/CT in breast cancer reported an improved preoperative localization of hot nodes (5). Subsequent studies confirmed the value of SPECT/CT for this purpose (6,7). Depicting SNs that are not visible on conventional images is helpful. There is a consensus that the axilla is the main basin for lymphatic drainage from the breast (8). However, SPECT/CT exposes additional sites of lymphatic drainage, including extra-axillary, which are not depicted by conventional scans. Depiction of the exact location of extra-axillary nodes facilitates the planning and execution of the surgery (9). There are certain atypical patterns, in which SNs are depicted in extra-axilla, such as the infra or superior clavicular regions, internal mammary nodes and other organs. In such an atypical pattern, it may sometimes become difficult to decide the extent of SNB. Thus, how atypical hot nodes are involved in lymphatic metastasis was investigated in the present study, particularly in level II (nodes lying behind the pectoralis major muscle in the axilla) to III (nodes lying above the pectoralis minor muscle in the infraclavicular region) regions.

Performing biopsies of SNs located in level II or III may be difficult with a small wound approach, in contrast to SNB in level I, which may normally be performed easily. National Comprehensive Cancer Network guidelines recommend that in the absence of gross disease in level II nodes, lymph node dissection should include tissue inferior to the axillary vein from level I to II, but regarding SNB, the sampling range is not mentioned in certain terms (10). In the process of SNB, where in particular nodes located in level II/III are much deeper than level I in the axilla, more widespread surgery may be unavoidable and it may take more time to complete SNB. In fact, SNB may be possible by forcing through the same wound into level II, in contrast to in level III. When multiple SNs were detected in the radioisotope method, the hottest node did not show metastasis. Thus, it is necessary to resolve a clinical question of how extensive the SNB can be performed, including level II/III in the axilla. Consequently, metastasis in level II/III was investigated, depending on whether hot nodes were depicted in level II/III on SPECT/CT.

## Material and methods

### Breast cancer patients

**Figure 1:**
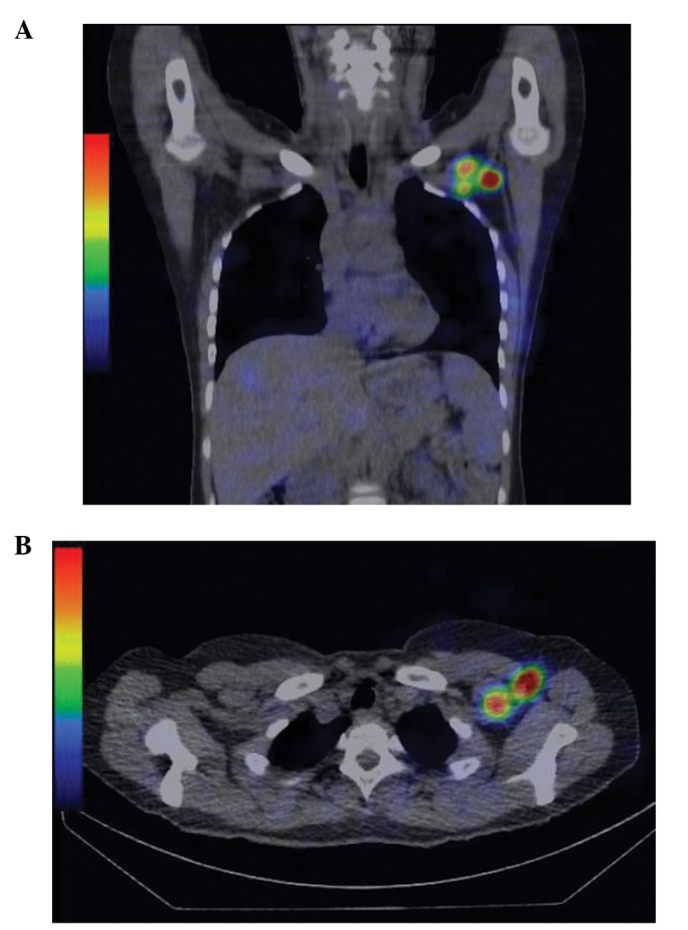
SPECT/CT scans of a patient in the left breast. Each shows the CT scan in the (A) coronal and (B) transaxial. In the fused images, the axillary SN is in color at levels I and II. SPECT/CT, single-photon emission computed tomography/computed tomography; SN, sentinel nodes.

Between June 2012 and August 2013, lymphatic flow was studied using planar lymphoscintigraphy and SPECT/CT in 92 clinical stage 0-IIA breast cancer patients. The axillary lymph nodes were classified by surgical description of ALND (level I, below and lateral to the pectoralis minor muscle; level II, behind the pectoralis minor muscle; and level III, above the pectoralis minor muscle) (3). The patients were divided into two groups: With or without hot nodes in level II/III on SPECT/CT, regardless of whether they were included in level I or not, and the existence of metastasis in level II/III was investigated as shown in Fig. 1. Hot nodes were observed in level II/III on SPECT/CT in 11 patients (12.0%) and no hot nodes were observed in 81 patients (88.0%). For identification of the risk factors, SNB-positive frequencies were evaluated through histology, estrogen receptor, progesterone receptor, human epidermal growth factor receptor type 2, nuclear grade, lymphatic invasion (ly), vascular invasion (v) and the existence of at least one hot node in level II/III, as observed on the SPECT/CT.

By contrast, SNB was revealed positive for metastasis in 12 patients (13.0%) and ALND was performed on all 12 patients. These 12 patients, on whom ALND was performed, were divided into two groups: With or without hot nodes in level II/III. The nodes in level II/III were harvested by ALND and the existence of metastasis was proven pathologically.

### SPECT/CT method and surgery

The SPECT/CT method and surgical procedure is as mentioned: A 2-day protocol was used with lymphoscintigraphy and SPECT/CT on the day 1 and the surgery occurring on day 2. This was followed by either mastectomy or a partial breast resection. SNB were performed during the same surgery. The day before surgery, ^99m^technetium-phytic acid was injected into two sites, at peritumor and around the nipple areola complex, subdermally and intradermally regarding depth in each site. The total radioactive dose was 74 MBq. SPECT acquisition (matrix 128^*^128, 24 sec/frame) was performed using 6° angular step, and a total acquisition time of 16 min and 7 sec (SPECT: 13 min 57 sec + CT: 2 min 10 sec). Static imaging in the supine position with simultaneous transmission scanning was performed 3 h after injection (Discovery NM/CT670; GE Healthcare, LLC, Princeton, NJ, USA). Following correction for attenuation and scatter, SPECT and CT axial 4.4-mm slices were generated and fused (Fujifilm RI Pharma Co., Ltd., Tokyo, Japan). Subsequently, SN mapping was performed to reveal hot nodes depicted by SPECT/CT (Fig. 1).

On day 2, radioisotope uptake in each lymph node was measured by a γ-probe using Neo-2000 (NeoProbe, Dublin, OH, USA) during surgery. Simultaneously, indigo carmine solution was injected similar to the ^99m^technetium-phytic acid injection. Blue nodes were found in the axillary sentinel lymph nodes intraoperatively. SNs were extracted and immediately examined by pathologists during the surgeries. When metastatic cancer was positively proven in the SN, ALND was performed, essentially up as far as level II, excluding isolated tumor cells.

### Statistical analysis

Statistical analysis between the two groups (with or without hot nodes in level II/III) was performed by the χ^2^ test. Univariate and multivariate logistic regression analysis was used for identification of SNB positive for metastasis risk factors. Using the χ^2^ test, a statistical analysis was undertaken between the two groups with or without hot nodes in level II/III, in 12 patients on whom ALND performed, the same as above. All the statistical analyses as mentioned above were performed with the JMP 10 software program (JMP 10.0.0 for Macintosh; SAS Institute, Inc., Cary, NC, USA).

## Results

### Patient characteristics

SPECT/CT was performed in 92 clinical stage 0-IIA breast cancer patients and the patient characteristics and sentinel node results are presented in Table I. The mean age was 59.9 years (35–81 years). A total of 43 patients had right breast cancer and 49 patients had left. The mean number of sampling lymph nodes in SNB was 1.75. And the mean number of hot nodes visualized on SPECT/CT was 1.49. Regarding SPECT/CT, all the patients had at least one hot node in level I, and there were no patients with SN in the internal mammary chain. In addition, regarding a group composed of 12 patients that had hot nodes depicted in level II/III on SPECT/CT, 10 had hot nodes in the two regions (level I and II), and two patients had hot nodes in all three regions (level I, II and III).

In patients with lymph flow, there was an SN in a single node field in 90 patients (97.8%) and two node fields in two patients (2.2%). In one patient, hot nodes were depicted in two node fields; axilla and liver, and in another patient the two node fields were depicted in the axilla and supraclavicular region.

Pathological lymph node metastasis was observed with a significantly higher frequency in the group with hot nodes depicted in level II/III than in the non-level II/III (Table I, P=0.014). In other clinical-pathological factors, there was a significant difference in ly (P<0.0001) and Ki67 (P=0.031), as shown in Table I.

In univariate analyses, hot nodes in level II/III, ly and Ki67 were all significantly associated with pathological SN metastasis (Table II, left column). In addition, using multivariate analysis, hot nodes in level II/III and ly were independent factors associated with metastasis pathologically found in axillary lymph nodes (Table II, right column). Hot nodes depicted in level II/III in SPECT/CT may be important preoperative information, as SN metastasis may be more strongly suspected.

By contrast, 12 SNB-positive patients on whom ALND was performed were investigated. In four of the 12 patients, the hot nodes were observed in level II/III on SPECT/CT, and no hot nodes in level II/III in eight patients, as shown in Table III. Of these four, there were two with hot nodes in level II with metastatic nodes pathologically evident in the same lesion (level II) and no metastasis in level III. However, there was pathologically no metastasis in level II/III among the eight patients who had no hot nodes in level II/III. Statistically there were no significant differences in the existence of metastatic nodes in level II/III (P=0.053). Notably, there were two cases in which metastasis was pathologically proven in SN at level II (not level III), where hot nodes were depicted in the same site (level II) on SPECT/CT.

## Discussion

SPECT/CT reveals various lymphatic drainages, including axilla, infra or superior clavicular region internal mammary nodes and other organs, while the axilla is the main basin for lymphatic drainage from the breast (4,8,9). These atypical patterns may sometimes make it difficult to decide the extent of SNB required.

Although axillary drainage is the principal lymphatic path of the breast, the breast lymphatic vessels drain to infraclavicular systems less frequently (8,11). The present study experienced SNs that could not be found in low-axilla where they typically exist, but were in a deeper area than observable through the surgeon’s sensation. Therefore, a hypothesis was developed that it may be useful in the process of intraoperative SNB to determine whether there are SNs in level II/III of axilla or not. By contrast, if lymphatic basin of extra-axillary areas is detected, this information may be helpful when considering systematic therapies. Uren *et al* (3) reported that at least one SN was observed in the axilla in the majority of patients, 96.7%. Additionally, the study described that an SN was observed at level II in the axilla in 10% of patients and an SLN at level III was observed in 2% of patients, using a peritumoral injection study. In the present study, there were hot nodes in at least one node in the axilla in all 92 patients, and a second hot node in level II/III in 11 patients (12.0%), indicating that this result is in accordance with a previous study (3).

Irregular locations of SNs have been reported, including in levels II/III or outside the axillary (12,13). Intradermal injections of tracer have been accepted to demonstrate the low frequency of accumulation in the internal mammary lymphatics (14). In the present results, no hot nodes were detected in internal mammary lymphatics, regardless of studies describing internal mammary lymphatic basins (15). Peritumoral and sub-areolar injection is preferable for the identification of the SNB (14,16), and peritumoral injections demonstrated a significantly lower proportion of patients who showed drainage to the internal mammary nodes (8). The two sites injection method; peritumor and around the nipple areola complex, was a large part of this study (77.2%). However, of note is the clinical inefficacy of the internal mammary node dissection as reported by Veronesi *et al* (17), indicating that there could be numerous problems surrounding the validity of internal mammary SNB (18). Thus, the present study focused on level I or II/III of axilla, with the exception of internal mammary chain.

With regards to the uncommon locations of SN, it was noted that a hot spot in the liver was observed in one case in the study, which may be explained as a direct lymphatic drainage route through the sub-diaphragmatic lymphatic plexus and abdominal wall vessels (Gerota’s para-mammary route) (19). Alternatively, Tanis *et al* (8) reported that the blockade of normal lymph flow can also cause drainage in a retrograde direction to the liver through the internal mammary chain. This atypical lymphatic basin could be depicted by SPECT/CT prior to surgery, therefore these irregular lymphatic basins may be an important source of information for determining a treatment strategy or surveillance following surgery, rather than during surgery (4).

In the present study, multivariable analysis indicated that the detection of hot nodes in level II/III on SPECT/CT and ly were significantly correlated with pathological metastasis in SN. Therefore, the present study demonstrated that the patients with a hot node in level II/III, as depicted by SPECT/CT, may have the risk of lymph node metastasis in the axilla. One clinical question regarding SNB in axilla would be how extensive are plural SNs that should be selected for SNB examination in those patients with SNs in level II/III in addition to in level I, and this has been largely unaddressed previously and remains unresolved to the best of our knowledge. With regards to this, the present study may provide evidence.

Additionally, in two of the four patients with hot nodes depicted as SN in level II/III by SPECT/CT, notably there were metastatic nodes pathologically evident in the same lesion in these two cases. Specifically, the two patients with hot nodes in level II had hot nodes present in level I in the axilla also, resulting in metastatic nodes pathologically evident in the same lesion (level II). This indicated that there may be a potential risk for metastasis in level II if hot nodes were observed in level II on SPECT/CT. Thus, if there were multiple SNs included in level II, the present data indicates that the SNs in level II should be sampled aside from SNs in the shallow level of axilla in level I, and excluding SN sampling in level II should be avoided as much as possible.

SNB is associated with morbidity less than ALND (21) and it is a prerequisite for reducing the requirement for ALND and avoiding the associated morbidity (22). In the results of a systemic review to investigate the safety of SNB without ALND of cN_0_ breast cancer patients, whilst the loco-regional recurrence rate was 0.6% for 36 months in no ALND with SNB-negative patients, the loco-regional recurrence rate increased to 1.7% (95% CI, 1.0–2.7) in non-ALND with SNB-positive patients indicating a threefold risk of recurrence (23). Thus, SNB without ALND may be unacceptable due to an increased risk for loco-regional recurrence in SNB-positive patients. The standards that followed the St. Gallen consensus meeting in 2013, ‘ACOSOG-Z011,’ affected numerous breast cancer surgeons. While the policy of avoiding full axillary clearance following 1–2 positive SNs was endorsed in situations of consecutive surgery and radiotherapy (73% YES and 21% NO), the completion of ALND was decided as safely avoidable in patients with 1–2 positive SNs and mastectomy without radiotherapy and this remains the large majority view (4% YES and 91% NO) (24,25). Presently, the prevailing belief is that local control is highly important with regards to surgery of the axilla, particularly in situations without application of radiotherapy.

The present data indicated that hot nodes in level II/III on SPECT/CT may be risk factors of SN metastasis, including SNs in the same site. In conclusion, a concept of local control in axilla is gaining recognition in breast cancer, and so it can be argued that whether there are hot nodes in level II/III in axillary as determined by SPECT/CT is important information. In particular support of this, patients observed with hot nodes in level II on SPECT/CT with metastasis were subsequently confirmed at the same site by pathological examination. Therefore, the risk of metastasis SNs in level II/III should be noted aside from those SNs in the shallow level of the axilla.

## Figures and Tables

**Table I:** Distribution of clinical and pathological characteristics among patients revealed pathologically metastasis in SN.

Patient characteristics	Total, n (n=92)	Pathologically positive for SNB, n (n=12)	Pathologically negative for SNB, n (n=80)	P-value
Age, years				
Mean (range)	59.9	57.5 (35–76)	60.3 (35–81)	NS
SD	12.4	11.9	12.5	
Main region of cancer				
A	27	1	26	NS
B	5	0	5	
C	38	6	32	
D	17	3	14	
E	5	2	3	
ER				
Positive	63	8	55	NS
Negative	24	4	20	
Unknown	5	0	5	
PgR				
Positive	51	6	45	NS
Negative	36	6	30	
Unknown	5	0	5	
HER2				
Positive	17	2	15	NS
Negative	68	10	58	
Unknown	7	0	7	
Nuclear grade				
1	36	4	32	NS
2	25	3	22	
3	24	5	19	
Unknown	7	0	7	
ly				
Positive	15	8	7	P<0.0001
Negative	70	4	66	
Unknown	7	0	7	
v				
Positive	6	0	6	NS
Negative	69	12	67	
Unknown	7	0	7	
Ki67, %				
Mean	25.3	41.2	22.8	P=0.031
SD	24.9	32.9	22.7	
Tumor size, cm				
Mean	1.86	2.58	1.74	NS
SD	1.38	1.29	1.37	
Pathology				
DCIS	20	0	20	NS
IDC	60	9	51	
Special type	12	3	9	
Hot nodes in level II/III				
Positive	11	4	7	P=0.014
Negative	81	8	73	

N, number of cases; ER, estrogen receptor; PgR, progesterone receptor; HER2, human epidermal growth factor receptor type 2; Ly, lymphatic space invasion; v, vascular space invasion; DCIS, ductal carcinoma *in situ*; IDC invasive ductal carcinoma; SD, standard deviation; NS, not significant. Bold value is statistically significant.

**Table II:** Univariate and multivariate logistic regression model of clinico-pathological factors and odds of metastasis pathologically found in axillary lymph node.

	Univariate analysis			Multivariate analysis		
		
	Odds ratio	95% CI	P-value	Odds ratio	95% CI	P-value
Ki67	0.11	0.83–9.28	0.031	0.65	0.047–10.77	0.75
Ly	4.78	4.78–87.74	<0.0001	24.78	4.77–197.40	0.00004
Hot node in Level II/III	5.21	1.166–21.68	0.024	8.89	1.129–88.91	0.042

Ly, lymphatic space invasion; OR, odds ratio; CI, confidence interval.

**Table III:** Distribution of the deepest metastatic lymph node examined pathologically among patients revealing hot nodes positive verses negative by SPECT/CT analysis.

Pathological findings	Metastasis in level I only, n	Metastasis up as far as level II, n	Total, n	P-value
SPECT/CT findings				
Hot nodes were detected in level II/III	2	2	4	
No hot nodes in level II/III	8	0	8	0.053
Total			12	

There were no patients exhibiting metastasis in level III.
